# Trans-Endplate Pedicle Pillar System in Unstable Spinal Burst Fractures: Design, Technique, and Mechanical Evaluation

**DOI:** 10.1371/journal.pone.0139592

**Published:** 2015-10-26

**Authors:** Chunfeng Zhao, Michio Hongo, Brice Ilharreborde, Kristin D. Zhao, Bradford L. Currier, Kai-Nan An

**Affiliations:** Biomechanics Laboratory, Division of Orthopedic Research, Department of Orthopedic Surgery, Mayo Clinic, Rochester, MN, 55905, United States of America; Klinikum rechts der Isar - Technical University Munich - TUM, GERMANY

## Abstract

**Background:**

Short-segment pedicle screw instrumentation (SSPI) is used for unstable burst fractures to correct deformity and stabilize the spine for fusion. However, pedicle screw loosening, pullout, or breakage often occurs due to the large moment applied during spine motion, leading to poor outcomes. The purpose of this study was to test the ability of a newly designed device, the Trans-Endplate Pedicle Pillar System (TEPPS), to enhance SSPI rigidity and decrease the screw bending moment with a simple posterior approach.

**Methods:**

Six human cadaveric spines (T11-L3) were harvested. A burst fracture was created at L1, and the SSPI (Moss Miami System) was used for SSPI fixation. Strain gauge sensors were mounted on upper pedicle screws to measure screw load bearing. Segmental motion (T12-L2) was measured under pure moment of 7.5 Nm. The spine was tested sequentially under 4 conditions: intact; first SSPI alone (SSPI-1); SSPI+TEPPS; and second SSPI alone (SSPI-2).

**Results:**

SSPI+TEPPS increased fixation rigidity by 41% in flexion/extension, 28% in lateral bending, and 37% in axial rotation compared with SSPI-1 (P<0.001), and it performed even better compared to SSPI-2 (*P*<0.001 for all). Importantly, the bending moment on the pedicle screws for SSPI+TEPPS was significantly decreased 63% during spine flexion and 47% in lateral bending (p<0.001).

**Conclusion:**

TEPPS provided strong anterior support, enhanced SSPI fixation rigidity, and dramatically decreased the load on the pedicle screws. Its biomechanical benefits could potentially improve fusion rates and decrease SSPI instrumentation failure.

## Introduction

Burst fracture in spine is an unstable fracture that often needs surgical stabilization with instrumentational fixation.[1–3] Numerous spinal internal fixation implants are available to stabilize the fracture segment using an anterior or a posterior approach.[4–6] The goal of any implant is to create and maintain reduction until spinal fusion occurs. Posterior surgeries are preferred by most surgeons because of the ease of exposure, decompression, reduction, fixation, and fusion. Short-segment pedicle instrumentation (SSPI) is commonly used for unstable burst fractures. [2, 7, 8]SSPI has enabled surgeons to instrument “one above, one below” the fractured vertebrae (2 motion segments), thus decreasing the exposure, preserving fusion levels, and making instrumentation removal less necessary. [9, 10] Unfortunately, such a construct puts higher loads on the pedicles and pedicle screws, which may lead to early implant failure due to pedicle screw bending or breakage or to bone failure with screw loosening or pullout. In the condition of unstable burst fractures with collapsed anterior and middle columns of the spine, physiologic loading is applied primarily to the instrumentation. The physiologic load at the thoracolumbar region is the greatest in the spine because of the large anterior moment arm. [11, 12] In the absence of load sharing, instrumentation failure and nonunion rates are greatly increased. Based on literature reports, instrumentation failure rate ranged from 25–60% and nonunion rate varied from 0–56% depending the approaches, graft material, and patient specific characteristics such smoking, overweight, diabetes, etc. [13–17] Biomechanical and clinical data have shown that anterior instrumentation with a graft presents some benefits in SSPI augmentation, including increasing the stiffness of the stabilized segment, increasing the rate of fusion, and decreasing the rate of implant failure. [6, 18] However, the additional anterior approach increases the morbidity and cost of the procedure substantially.

The purpose of this study was to design and biomechanically test a new device that would be used for posterior approach, named the Trans-Endplate Pedicle Pillar System (TEPPS), to reinforce the anterior column with SSPI. The device can be used with currently available pedicle screw /rod systems. The goal of the device is to reduce implant failure and loss of correction without extending fixation segments and obviating a secondary anterior approach. We hypothesized that this TEPPS anterior supporting system would strengthen the SSPI by decreasing spine segmental motion and the loading on the pedicle screw compared to the SSPI fixation alone using a human cadaveric model.

## Materials and Methods

### Specimen Preparation

Following Mayo Clinic Institutional Review Board approval, six fresh-frozen adult human cadaver spines consisting of 4 motion segments, T11 to L3, were used to test the TEPPS vs SSPI. Written informed consent from the donors was obtained for the use of the samples in research. The specimens were from 3 female adults and 3 male adults with an average age of 76.7 years (range, 63–84 years). The specimens were screened radiographically to exclude those with occult spinal disorders. After the spines were harvested, the ribs, muscle, and adipose tissues were removed from each specimen, leaving ligamentous structures and joint capsules intact. For fixation, Kirschner wires and screws were passed into the T11 and L3 vertebral bodies. The superior half of the T11 and the inferior half of the L3 vertebral bodies were then potted in aluminum molds using polymethyl methacrylate (HYGENIC, Coltene/Shaledent Inc. Cuyahoga Falls OH) for the subsequent procedures of our study, as detailed below.

### Unstable Burst Fracture Creation and SSPI Fixation

**Fig 1:**
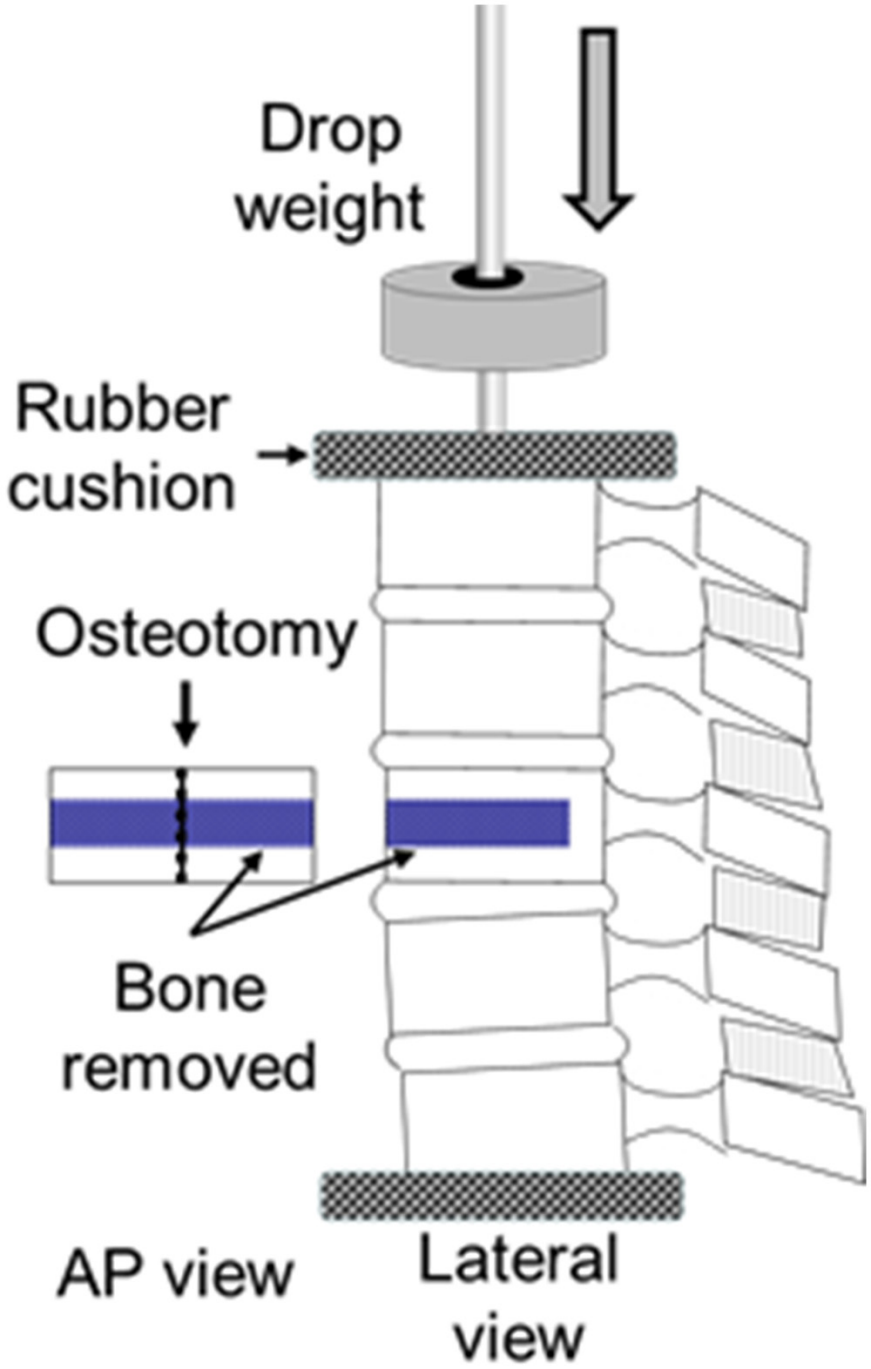
L1 fracture illustration shows that a middle portion of the L1 vertebral body was removed and a vertical osteotomy was made to create defect of the L1 for the fracture model. A dead weight was dropped to the top of T11 with a rubber cushion protection to prevent direct impact on the T11 causing fracture.

**Fig 2:**
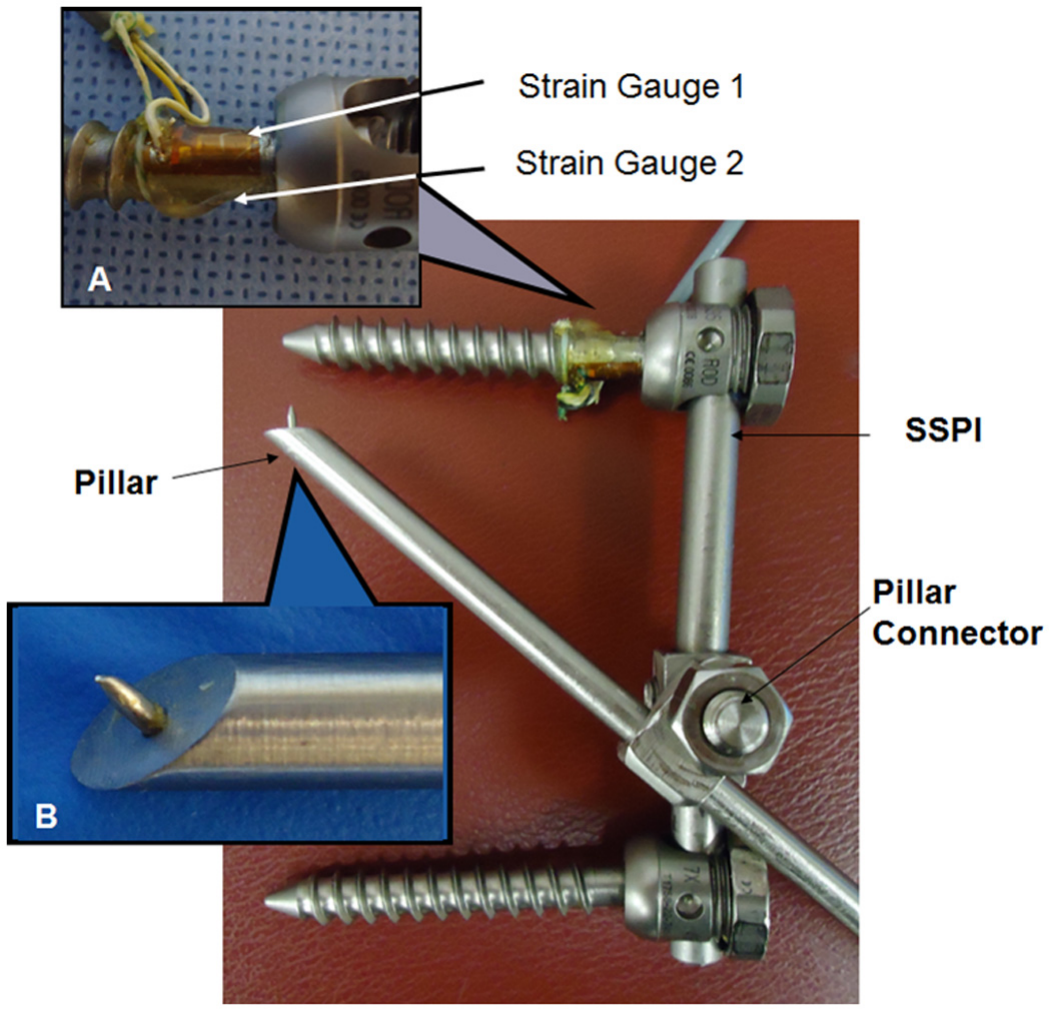
SSPI (Moss Miami System) combined with Trans-Endplate Pedicle Pillar System (TEPPS). **A**, A pair of strain gauges mounted on the pedicle screw measures the screw bending moment. **B**, The pillar tip has a pin-foot that can be anchored into the endplate to avoid pillar sliding. The pillar was connected to the SSPI through a pillar connector.

The unstable vertebral fracture was created by removing the middle portion (about one-third) of the L1 vertebral body, leaving the posterior wall intact. Additional weakening of vertebral support was achieved by performing a vertical osteotomy on both endplates and on the posterior wall of L1 (Fig 1 within square box). Then, per the established spine fracture creation protocol, an incremental weight drop impact was carried out on top of the cap, starting with 3 kg dropped from a constant height of 1.4 meters.[19] Combined with the osteotomy, all cadaver spines had an unstable burst fracture created with a weight drop of 10 kg (Fig 1). L1 fracture was confirmed by X-ray (Siremobil Compact, Siemens, Munich Germany), and no adjacent vertebral body fractures were observed. Following L1 fracture creation, posterior SSPI was carried out using the Moss Miami System (DePuy Spine Inc, Raynham, Massachusetts). A pair of strain gauges (Measurement Group, Inc. Raleigh, North Carolina) were mounted in opposing sides on the base of a pedicle screw in order to measure the force applied to the screw during mechanical testing (Fig 2A). Force-measurable screws were only used bilaterally at the upper vertebrae (T12) and not used for the lower vertebrae (L2). The pedicle screws (7 mm in diameter) were inserted in the adjacent vertebrae above and below the injured level in a standard manner. The force-measurable screws were inserted with the strain gauges aligned vertically to the spine cross section, so that the bending moment could be measured and calculated during spine motion. Before force-measurable screw insertion, the strain gauge was calibrated by applying varied dead weights on the silicon extension bar of the pedicle screw with a distance 10 cm to the strain gauge. The bending moment was calculated with known weight multiplied the distance between the strain gauge and the applied force point (10 cm). The equation obtained from linear regression was used for the experimental screw bending calculation. Four pedicle screws were then connected with 2 rods (6.35 mm in diameter). Before TEPPS insertion, the fracture was reduced in the desired physiologic position, under fluoroscopic guidance.

### Technique of Pillar Insertion

**Fig 3:**
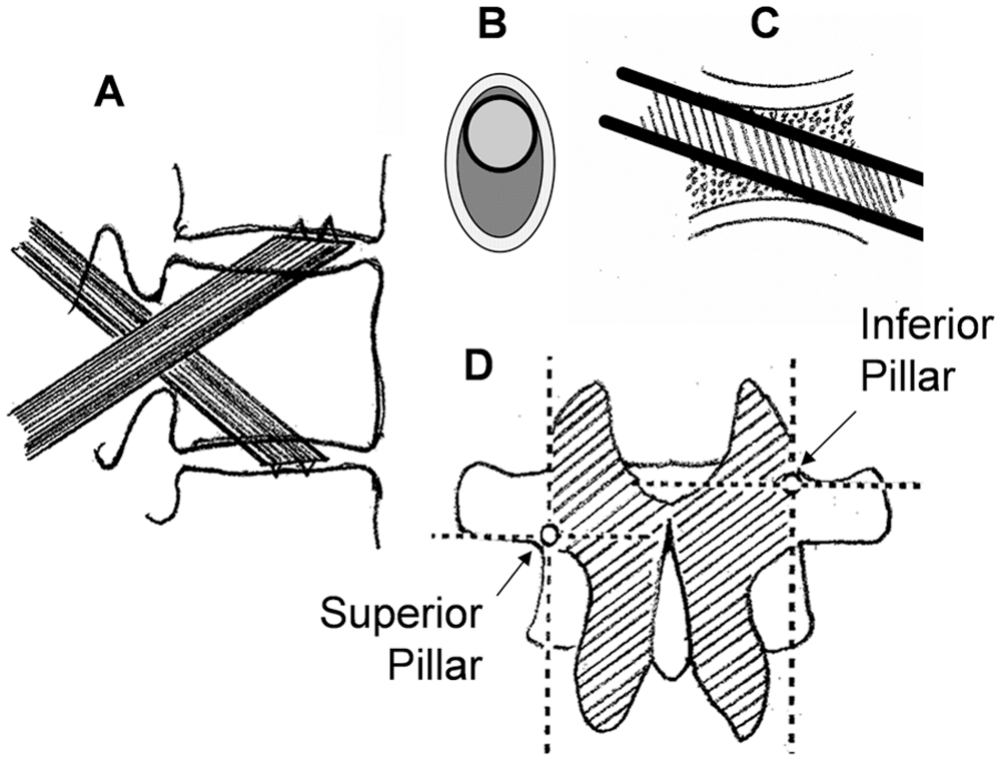
Pillar Insertion of the Trans-Endplate Pedicle Pillar System. **A**, Spine sagittal plane of the pathway of the pillar insertion. **B**, Cross section of the pedicle (coronal plane) shows its oval shape. **C**, Longitudinal section of the pedicle and pillar after insertion. **D**, Pillar insertion landmarks: inferior pillar (right) was advanced obliquely from the intersection of the vertical line at the lateral border for the superior articular process with the horizontal line at the upper margin of the transverse process, and the superior pillar was inserted obliquely from the intersection of the vertical line at the lateral border for the superior articular process with the horizontal line at the low margin of the transverse process.

The TEPPS includes 2 pillars and 2 connecters that attach to the rods. The trans-endplate pillar (TEP) is a smooth cylindrical rod, 5 mm in diameter. The tip of the pillar is formed in an oblique plane (45°) with a tooth in the center to anchor it in the endplate during insertion (Fig 2B). A connector was designed to affix the pillar to the rod (Fig 2). The pillar passes obliquely through the pedicle of the fractured vertebral body from the posterior approach, crosses the endplate of the fractured vertebra and docks on the adjacent endplate (Fig 3A). In the coronal plane, the pedicle has an oval shape (Fig 3B). The vertical diameter of the pedicle is larger than its transverse diameter, which provides space within the pedicle for a screw, or pillar, to be inserted at various angles in the sagittal plane. Furthermore, in the sagittal plane, the pedicle is short, with a narrow midsection and larger, funnel shaped portions at the junctions with the articular facets posteriorly and the vertebral body anteriorly (Fig 3C). These anatomical characteristics allow the pillar to pass through the pedicle obliquely without causing a fracture. Indeed, in standard pedicle screw insertion, accidental penetration of the endplate by a pedicle screw has been reported.

**Fig 4:**
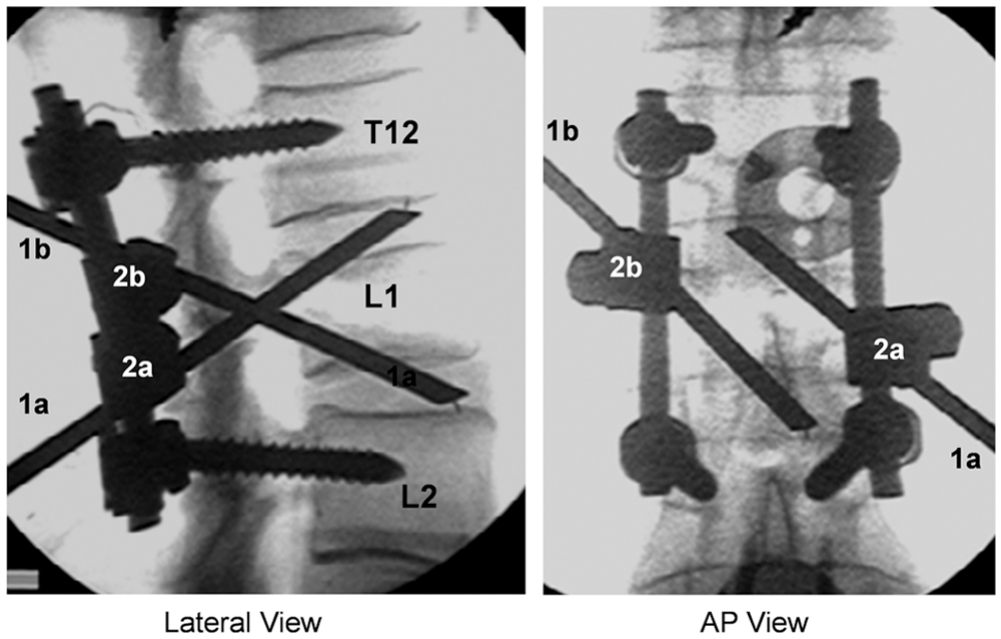
Radiograph of SSPI+TEPPS (Moss Miami System Combined With Trans-Endplate Pedicle Pillar System). Radiograph of fractured spine in L1 reveals SSPI+TEPPS instrumentation. Left, Lateral view shows the upper endplate pillar (1a) with its connector (2a) and the lower endplate pillar (1b) with its connector (2b). Right, Anteroposterior view shows the upper endplate pillar (1a) with its connector (2a) and the lower endplate pillar (1b) with its connector (2b).

Similar to the pedicle screw insertion technique, a pillar rod can be inserted through a pedicle in a caudal or cranial direction. The superior TEP, which is orientated from caudal posterior to cranial anterior, penetrates the upper endplate of the fractured vertebral body and is levered up on the lower endplate of the upper adjacent vertebra. The entrance point of the superior TEP is identified by the intersection of a vertical line at the lateral border of the superior articular process with a horizontal line at the lower margin of the transverse process. The inferior TEP enters from the site at the intersection of the same vertical line as the superior TEP insertion with the upper margin of the transverse process (Fig 3D). The inferior TEP is advanced obliquely from the entrance toward the lower endplate of fractured vertebra and is docked on the upper endplate of the lower adjacent vertebra (Fig 3A). In our study, each pillar was inserted under fluoroscopic guidance until the tip of the pillar touched the endplate. After TEP insertion, the connectors were tightened to connect the TEP to the SSPI rods (Figs 2 and 4).

### Mechanical Testing

**Fig 5:**
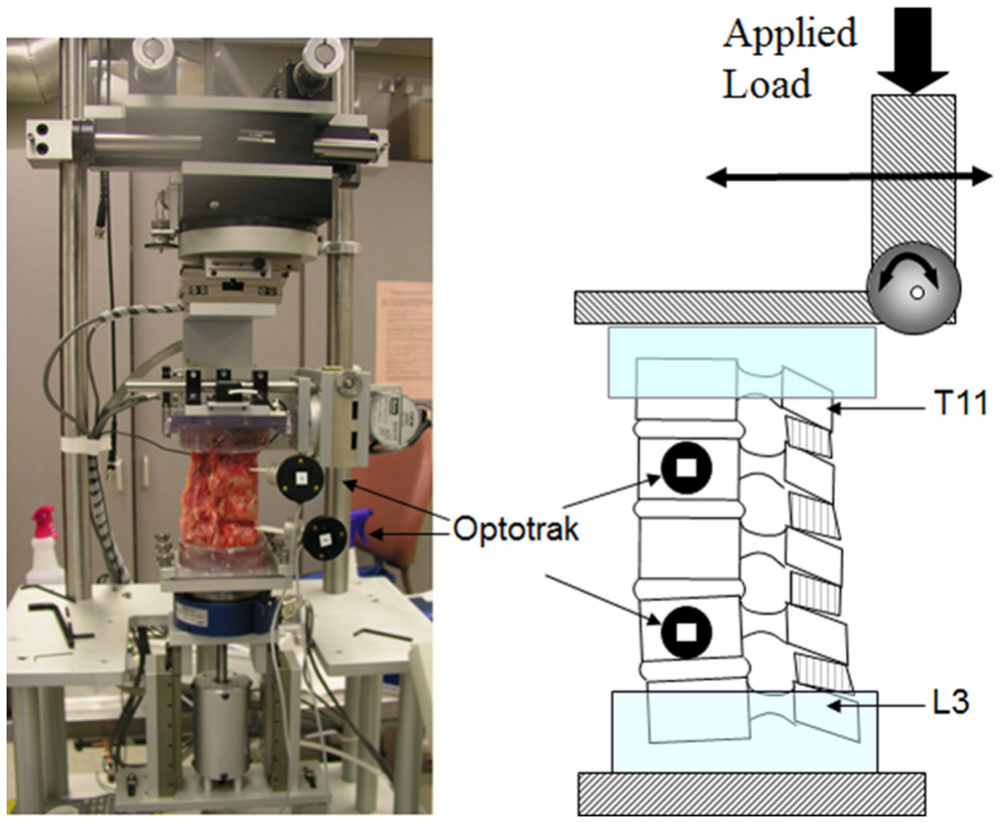
Custom-Made Spine Testing Apparatus. A 7.5-Nm moment was applied to the spine during flexion/extension, lateral bending, and axial rotation. Segmental motion was measured using the Optotrak Certus video-based data acquisition system (Northern Digital Inc, Waterloo, Ontario, Canada).

The potted specimens of the T11 and L3 vertebral bodies were mounted for spine testing to a custom-made device described previously.[20] Briefly, the spine-testing device uses pneumatic methods with load control to test 2 passive axes of translation in the transverse plane and 1 distal axial stage. Flexion-extension, lateral bending, and axial rotation motions are generated by 3-axis gimbals and stepper motors (Fig 5). Forces and moments are measured with a 6-component load cell (JR3 Inc, Woodland, California). Pure moment loading is achieved by eliminating shear loads with linear slides, which is validated by force and moment profiles during testing. Motion is induced in flexion-extension, lateral bending, and axial rotation with continuous cycles under pure moment to 7.5 Nm at 3° per second. The 7.5-Nm load is similar to that used in lumbar testing protocols.[21, 22] Each load is applied for 4 cycles to precondition the spine before analysis of data from a fifth cycle.

**Fig 6:**
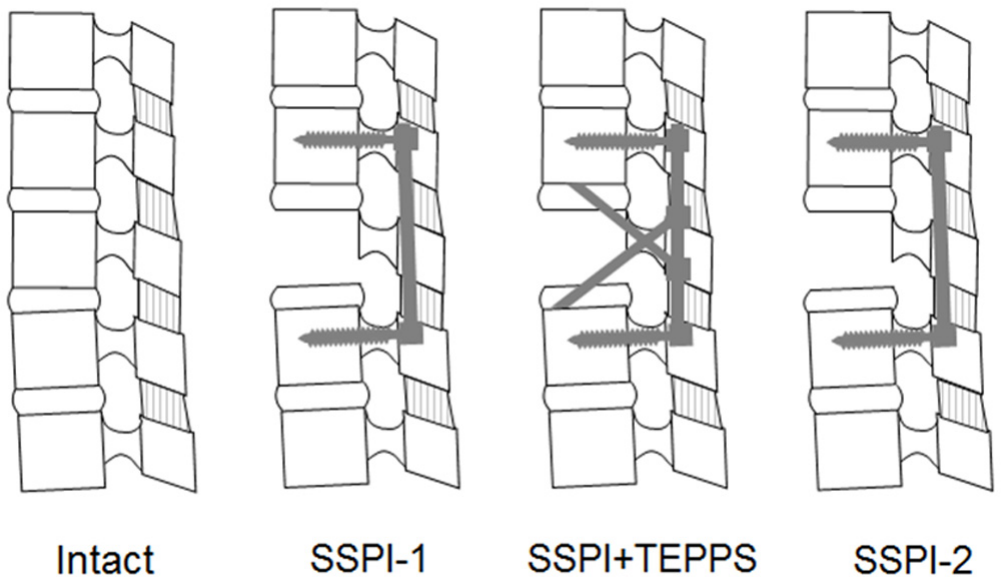
Sequence of the 4 Test Conditions. From left to right: 1) intact spine; 2) fractured spine with SSPI fixation (SSPI-1); 3) fractured spine with SSPI+TEPPS (Moss Miami System combined with Trans-Endplate Pedicle Pillar System) fixation; and 4) fractured spine with SSPI fixation again with pillar withdrawn (SSPI-2).

Kinematic measurements between T12 and L2 were obtained at a frequency of 150 Hz using an Optotrak Certus video-based data acquisition system (Northern Digital Inc, Waterloo, Ontario, Canada) and accompanying software (MotionMonitor, Innovative Sports Training Inc, Chicago, Illinois) (Fig 5). The force applied to the upper 2 pedicle screws was also measured by the strain gauge mounted on the pedicle screws using a technique described previously. [23, 24] Each specimen was tested in a fixed testing sequence under 4 different conditions: condition 1 (intact spine); condition 2 (first test of SSPI alone after fracture creation [SSPI-1]); condition 3 (SSPI+TEPPS); and condition 4 (second test of SSPI alone [SSPI-2] with pillars withdrawn) (Fig 6). Since the fractured spine condition was quite unstable, the fractured spine without the instrumentation condition was not tested.

### Statistical Analysis

Mean and standard deviation in angular motion of flexion/extension, lateral bending, and axial rotation in intact spine, SSPI-1, SSPI+TEPPS, and SSPI-2 were used to summarize the spine kinematic data. The peak moment on the 2 upper pedicle screws was averaged and mean and standard deviation were summarized for analysis. As the sample size was mall, after consulting with a biostatistician, a non-parametric repeated measure ANOVA, Friedman’s procedure statistics, was used to compare values from the first test (intact) to the last test (SSPI-2). All analyses were performed using SAS version 9.3 software (SAS Institute Inc, Cary, North Carolina). Any *P* value less than .05 was considered to be statistically significant.

## Results

**Fig 7:**
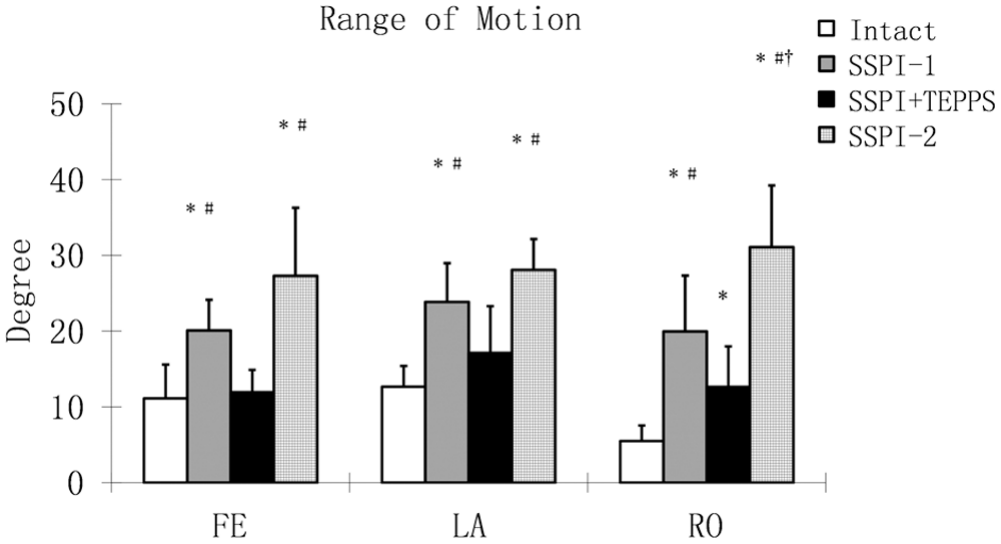
Total Range of Motion. The mean peak spine motion from T12 to L2 in flexion/extension, lateral bending, and axial rotation for each of the 4 test conditions (intact; first test of SSPI-1 [Moss Miami System] alone; SSPI+TEPPS [Moss Miami System combined with Trans-Endplate Pedicle Pillar System]; and second test of SSPI-2 alone after removal of pillar). Asterisk (*) indicates a significant difference from intact spine (*P* < .001); pound sign (#) indicates a significant difference from SSPI+TEPPS (Moss Miami System combined with Trans-Endplate Pedicle Pillar System) fixation (*P* < .001); and dagger (†) indicates a significant difference from SSPI-1 (first test of SSPI System alone) (*P* < .001).

The total range of motion (ROM) of flexion/extension, lateral bending, and axial rotation in the intact spine and SSPI+TEPPS were significantly less than that in both SSPI-1 and SSPI-2 (*P* < 0.001). There was no significant difference between the intact spine and SSPI+TEPPS in flexion/extension and lateral bending. However, a larger axial rotation was observed in SSPI+TEPPS compared to that in the intact spine (*P* < 0.001). The fracture segmental motion fixed with SSPI+TEPPS compared to that in SSPI1 decreased 41% in flexion/extension, 28% in lateral bending, and 37% in axial rotation. The ROM of SSPI-1 in all 3 dimensions of motion (flexion/extension, lateral bending, and axial rotation) was significantly lower than that for the SSPI-2 (*P* < 0.0015) (Fig 7).

**Fig 8:**
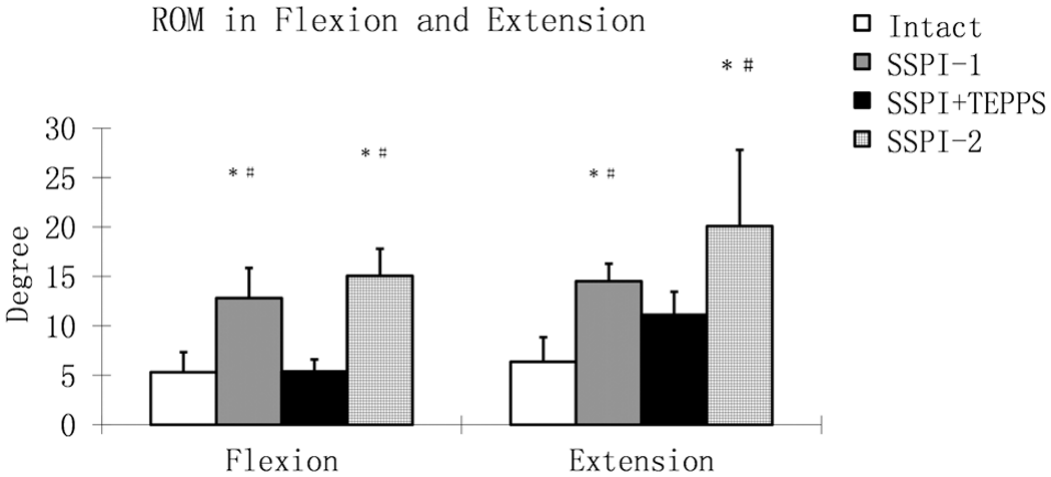
Range of Motion in Flexion and Extension. Mean of peak spine motion from T12 to L2 in flexion and extension for each of the 4 test conditions: (intact; first test of SSPI-1; SSPI+TEPPS; and second test of SSPI-2 alone). Asterisk (*) indicates a significant difference from the intact spine (*P* < .001), and pound sign (#) indicates a significant difference from SSPI+TEPPS (*P* < .001). For expansion of abbreviations, see Fig 6 legend.

Since we had speculated that TEPPS would effectively share axial compressive loading, the flexion /extension motion was isolated for analysis. The flexion ROM in intact spine and in SSPI+TEPPS under a 7.5-Nm moment was significantly lower than that in SSPI-1 and SSPI-2 (*P* < 0.0015). There was no significant difference in flexion ROM between intact spine and SSPI+TEPPS. SSPI-2 showed greater flexion compared to that in SSPI-1 (*P* < 0.001). In extension motion, the ROM of intact spine and SSPI+TEPPS was significantly lower than that of both SSPI-1 and SSPI-2 (*P* < 0.001). However, there was no significant difference between intact spine and SSPI+TEPPS, or between SSPI-1 and SSPI-2 (Fig 8).

**Fig 9:**
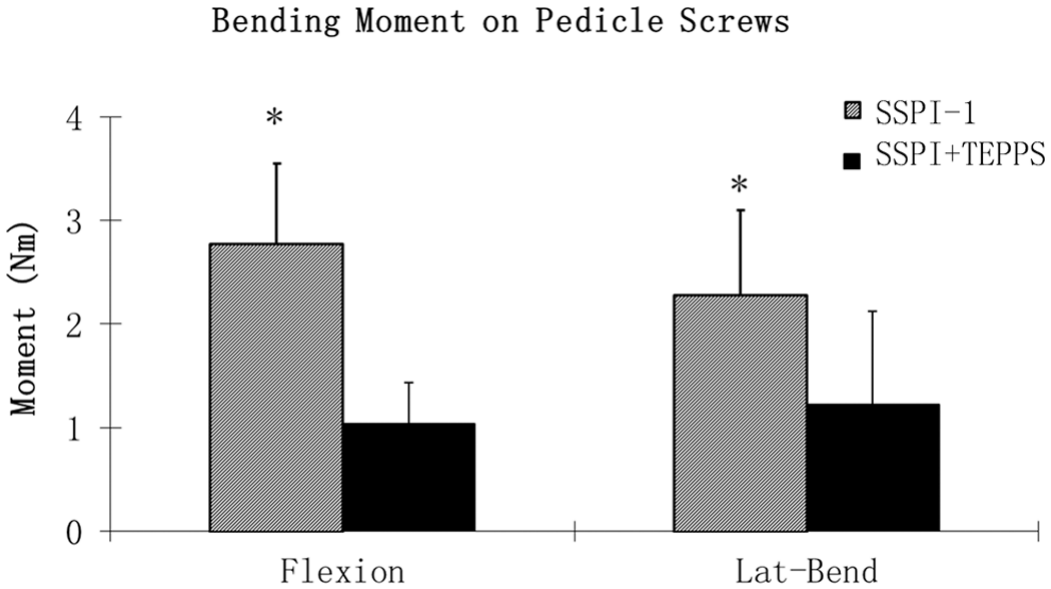
Bending Moment on Pedicle Screws During Flexion and During Lateral Bending. Mean peak moment applied to pedicle screws during flexion and lateral bending. Asterisk (*) indicates a significant difference from intact spine (*P* < .001). For expansion of abbreviations, see Fig 7 legend.

Since the strain gauges mounted on the pedicle screws were placed sagittally during screw insertion, only sagittal plane bending moment was measured. Therefore, the strain gauge data were analyzed only for spine flexion and for lateral bending. The bending moment on the pedicle screws in SSPI+TEPPS decreased 63% during flexion and 47% during lateral bending compared to that in SSPI-1, which was a statistically significant finding (*P* < 0.001) (Fig 9).

## Discussion

The current study was to address a clinical important issue, e.g. instrumentation fatigue failure, which is one of the common complications following SSPI in spine surgery. The most common failure modes include screw bending or breakage occurs in the dense bone of young trauma patients [25–27] or pedicle screw loosening, toggling, or pullout in the osteoporotic bone in older patients.[26, 28, 29]. The use of an anterior strut bone graft with or without anterior instrumentation to reduce the bending moment to the posterior instrumentation is supported by the findings of numerous biomechanical studies.[18, 30, 31] Gurwitz et al [32] investigated the stiffness in 3 surgical approaches for lumbar burst fracture using short-segment posterior instrumentation with or without an anterior instrumentation and bone strut. Their results suggest that SSPI alone cannot restore the degree of stiffness or rigidity in the injured spine to that in the intact spine. Although SSPI combined with anterior instrumentation with or without anterior strut grafts restored the spine stability back to normal in flexion/extension and lateral bending motion, but did not restore the torsional rigidity of the injured spine. They therefore recommend possible external bracing to provide additional external rotational support. Our data also showed that the SSPI alone did not restore the spine stability of the fractured spine to normal in all dimensional motion. However, combining SSPI with TEPPS increased torsional rigidity about 30% to 40% in flexion/extension, lateral bending, and axial rotation. In particular, the increase in fracture rigidity was about 60% in flexion alone, which is considered to be the major motion causing instrumentation failure and recurrence of kyphosis. Whereas SSPI+TEPPS still presented larger motion in axial rotation compared to that in intact spine, it did restore the stability in flexion/extension and in lateral bending compared to that in intact spine. Therefore, the fractured spine stability restored by SSPI+TEPPS was very comparable to the SSPI combined with anterior instrumentation reported by Gurwitz et al. [32]

Sagittal bending moment applied to the pedicle screw during spine motion has been studied using strain gauges mounted on the pedicle screw.[23] Using human cadavers, Chiba et al [24] studied SSPI for burst fractures. Under a 5-Nm flexion torque applied to the spine, a 1.2-Nm to 1.7-Nm moment was measured for each pedicle screw when SSPI alone was used to fix the spine. Augmented offset laminar hooks were then added to the SSPI above the superior pedicles and below the inferior pedicles, which decreased the bending moment applied to the pedicle screws about 50%. However, these offset hooks require extended spine segment for fixation and fusion. Our data showed that the flexion bending moment in SSPI alone was 2.7 Nm, which was more than double that reported by Chiba et al, which may be due to the larger torque (7.5 Nm) applied to the spine during our study. In addition, the strain gauges in our study were mounted on the base (close to the rod) of the screw shank rather than within the pedicle screw through a canal as in Chiba et al’s study. Therefore, the moment arm applied to the pedicle screw in our study might be longer than that in Chiba et al’s study. Furthermore, our strain gauge mounting also prohibited full insertion of the pedicle screw, which caused a high bending moment on the screw. Clinical data have shown that pedicle screw breakage often occurs at the base, which is exposed outside the bone where there is the largest bending moment.[28, 33–35] Our strain gauges were mounted at this level. Nevertheless, the use of our novel device (TEPPS) decreased the screw bending moment more than 63% without requiring extension of the fixation segment.

The technique of TEPPS avoids an anterior approach, but accurate pillar insertion is critical, which requires a more precise technique than that used in regular pedicle screw insertion. Pedicle screw misplacement has been widely reported, [36, 37] with a risk of spinal cord or nerve root injuries. Since the pillar is inserted caudally or cranially to penetrate the endplate of the fractured vertebra, the pillar diameter should be smaller than a pedicle screw that can be used in the pedicle. In the current study, the pillar diameter was 5 mm compared to the 7-mm diameter of the pedicle screws. During our experimental procedure, the pillars were all easily and successfully inserted within the pedicle under fluoroscopic guidance. However, surgical standardization of pillar insertion and safety issues should be further addressed in detail on the basis of spine anatomy related to pillar diameter and insertion angle in 3 dimensions.

A 2007 report by Mahar et al [38] introduced a novel technique to improve SSPI rigidity by using pedicle screw fixation at the fractured vertebral body. Their biomechanical data revealed that this reinforced SSPI procedure increased the stiffness in axial rotation but not in flexion/extension or lateral bending. However, their clinical data showed that 9 patients treated with this technique had an average correction loss of 5.4° at 4.4-month follow-up. This finding indicated that the pedicle screws applied at the injury level could have some benefits for SSPI fixation, but these extra pedicle screws did not provide anterior load sharing and rigidity for flexion and lateral bending. Our newly designed device (TEPPS) not only enhanced the rigidity in all 3 dimensions of motion, but also provided sufficient anterior load sharing to effectively decrease the likelihood of instrumentation failure and loss of correction.

Our study had some limitations and unknown factors that may require further investigation. First, the specimens used in the study were all elderly spines with an average age of 76 years, which is older than most patients who require surgery for a burst fracture ranged 20 to 50 years old. [1, 2, 7]We also did not check bone density (DXA) for the specimens. Second, the superior 2 pedicle screws could not be fully inserted due to the strain gauges mounted on them, which is not clinically relevant. This impediment might not only cause the screw bending moment to be increased but might also affect screw fixation rigidity, which was indicated by the larger motion of the SSPI-2 compared to that of the SSPI-1. Although the testing sequence was a factor, the SSPI+TEPPS was always tested after the SSPI-1 and still showed more rigidity than the SSPI-1page. Therefore, this fixed testing sequence won’t alter the conclusion of our results. Third, the location of the pillar tip standing on the endplate may result in its penetration of the endplate. This was not observed in any of the specimens by fluoroscopy or after testing by direct observation. However, our study was not performed with fatigue testing that the pillar could penetrate the endplate under fatigue loading in clinical scenario. The risk of pillar penetration, especially in osteoporotic spine, could be reduced by cannulating the pillar in future designs. Bone cement could be injected through the pillar and placed at the end of the pillar to increase the hard surface area. Fourth, since each pedicle can hold only 1 pillar, we elected to place 1 pillar facing up and one pillar pointing down. Although this pillar insertion was asymmetric, each pillar was within the immobilized spine segment and we did not observe any subsequent abnormal motion of the spine. We did not study other options of pillar insertion, such as placing both pillars directed cranially or caudally. Finally, our sample size was relatively small. However, significant differences were obtained with appropriate statistical methods due to large changes in both spine rigidity and screw bending moment before and after pillar insertion.

In summary, the device (TEPPS) we designed is able to join with a SSPI system to enhance spine fixation rigidity and decrease the pedicle screw bending moment. SSPI was used to study the effectiveness of TEPPS in a burst fracture model using human cadavers. We found TEPPS to significantly increase the SSPI fixation rigidity in all directional motions, with the most improvement in flexion. More importantly, the pedicle screw bending moment dramatically decreased by more than 60% after TEPPS application. TEPPS could also be adopted for other SSPI systems and not be limited just to SSPI. We believe that combining TEPPS with SSPI would improve clinical outcomes for unstable burst fractures by improving spine stability and decreasing instrumentation-associated complications.
